# The Arachidonate 15-Lipoxygenase Enzyme Product 15-HETE Is Present in Heart Tissue from Patients with Ischemic Heart Disease and Enhances Clot Formation

**DOI:** 10.1371/journal.pone.0161629

**Published:** 2016-08-23

**Authors:** Annika Lundqvist, Mikael Sandstedt, Joakim Sandstedt, Ruth Wickelgren, Göran I. Hansson, Anders Jeppsson, Lillemor Mattsson Hultén

**Affiliations:** 1 Department of Clinical Chemistry and Transfusion Medicine, Institute of Biomedicine, Sahlgrenska Academy, University of Gothenburg and Department of Clinical Chemistry, Sahlgrenska University Hospital, Gothenburg, Sweden; 2 Translational Sciences, AstraZeneca R&D, Mölndal, Sweden; 3 Department of Cardiothoracic Surgery, Sahlgrenska University Hospital Gothenburg, Sweden and Department of Molecular and Clinical Medicine, Institute of Medicine, Sahlgrenska Academy, University of Gothenburg, Gothenburg, Sweden; 4 Wallenberg Laboratory, Department of Molecular and Clinical Medicine, Institute of Medicine, Sahlgrenska Academy, University of Gothenburg, Gothenburg, Sweden; Southern Illinois University School of Medicine, UNITED STATES

## Abstract

Ischemic heart disease is a major cause of death and morbidity and the search for novel therapeutic targets is still required. We have previously shown that the enzyme arachidonate 15 lipoxygenase (ALOX15), which catalyzes the conversion of arachidonic acid to 15-hydroxy eicosatetraenoic acid (15-HETE), is highly expressed in ischemic heart tissue, but its role in the pathogenesis of ischemic heart disease is unclear. Here we showed that expression of ALOX15, but not ALOX12 or ALOX15B, was increased in ischemic versus non-ischemic human heart biopsy samples. A similar ALOX expression pattern was found in hypoxic human cardiomyocytes and cardiac endothelial cells. We also showed that levels of 15-HETE were significantly higher in ischemic versus non-ischemic human heart biopsy samples and showed a tendency to increase in serum from the patients with ischemic heart disease. Moreover, hypoxia increased the production of 15-HETE levels from human cardiomyocytes and cardiac endothelial cells. The hypoxia-induced increase in 15-HETE levels from human cardiomyocytes was inhibited by the ALOX15 inhibitor baicalein. Finally, by using intrinsic rotational thromboelastometry, we showed that human whole blood clotted faster in the presence of 15-HETE. In summary, we propose that increased ALOX15 expression in heart tissue under ischemic conditions may lead to increased production of 15-HETE, potentially contributing to thrombosis.

## Introduction

Ischemic heart disease is one of the most common causes of death and morbidity worldwide. While important advances in clinical therapeutics have been made during the past century, prediction, prevention and treatment of adverse cardiovascular events—such as acute myocardial infarction—need to be improved further. Possible therapeutic targets include enzymes involved in lipid signaling and metabolic pathways. One such example is 15-lipoxygenase (ALOX15), which is present in the human myocardium [1, 2] and we have previously shown that its expression is increased in ischemic heart tissue [2]. Furthermore, the structurally related enzyme ALOX15B, which is increased in hypoxic human macrophages and symptomatic atherosclerotic carotid plaques [3, 4] is known to associate with a history of atherothrombotic events [5].

ALOX15 and ALOX15B catalyze the conversion of arachidonic acid to hydroperoxy derivatives such as 15-hydroxyeicosatetraenoic acid (15-HETE) [6], which have been implicated in many physiological and pathological processes [7] and thus are possible mediators for the effects of ALOX15/ALOX15B. In support of this, a recent biomarker study reported increased levels of HETEs in the plasma of patients with ischemic stroke [8]. Furthermore, we and others have previously shown that 15-HETE potentiates platelet aggregation, and that it is elevated in pathologic states associated with platelet hyperfunction [9, 10]. In addition, a recent study in a rat model of pulmonary hypertension showed that hypoxia increased coagulation and thrombosis, and the effects of hypoxia were reduced by inhibition of ALOX15 [11]. However, the role of ALOX15 in the pathogenesis of ischemic heart disease remains unknown.

Here we investigated whether increased ALOX15 expression and 15-HETE production could potentially contribute to ischemic heart disease by analyzing 15-HETE concentrations in heart tissue and serum from patients with and without ischemic heart disease. In addition, we assessed the effect of hypoxia on ALOX15/15-HETE production in human-derived cardiomyocytes and cardiac endothelial cells, which account for 30% and 60% of the total number of cells in the heart, respectively [12].

## Material and Methods

### Human material

Cardiac tissue from the right atrium and blood samples were obtained from patients undergoing aortic valve replacement (AVR, n = 5) or coronary bypass grafting (CABG, n = 5). Heart tissue samples were immediately placed in RNAlater solution (Qiagen, Valencia, CA) for further analysis by quantitative real-time PCR (RT-qPCR) according to the manufacturer’s instructions. For control samples, we purchased total RNA from right atrium from normal tissue from 3 human adults (Invitrogen and BioChain, CA).

Ethical approvals for the described human studies were obtained from the Ethical Committee of the University of Gothenburg. Written informed consent was obtained for all subjects prior to inclusion in the study.

### Cell culture

Human cardiomyocytes derived from pluripotent stem cells were purchased from Cellartis, Takara Clontech, Gothenburg, Sweden. The cardiomyocytes had been differentiated for 20 days in serum-free medium according to the manufacturer’s protocol, and shown to have a gene expression profile similar to that of adult cardiomyocytes and human heart tissue [13, 14]. We cultured the cells for 4 days before starting the experiments and observed that they were beating spontaneously after one day of culture and throughout the experiment. To show that cells expressed cardiomyocyte-specific markers, we stained the cells with anti-cardiac troponin I antibody (ab47003, Abcam, Cambridge, UK), phalloidin, a high-affinity F-actin probe (Sigma-Aldrich, St Louis, MO) and 4′,6-diamidine-2′-phenylindole dihydrochloride (DAPI) (Sigma-Aldrich) (S1 Fig).

Human cardiac endothelial cells were purchased from PromoCell, PRO C-12285, MEDIQIP AB, Huddinge, Sweden, and cultured according to the manufacturer’s protocol. The cells expressed the endothelial cell marker CD31 (ab 303101, BioLegend, San Diego, CA) (S2 Fig).

To mimic the effect of ischemia, cells were incubated under normoxic (21% oxygen) or hypoxic (1% oxygen) conditions for 6 h. Baicalein, a 12/15-lipoxygenase inhibitor, was from Sigma-Aldrich.

### Analysis of gene expression

Expression of human ALOX12, ALOX15, and ALOX15B mRNA was determined using quantitative RT-qPCR. Total RNA was extracted from human heart biopsies and cultured human cardiomyocytes and cardiac endothelial cells using RNeasy Kit (Qiagen, Hilden, Germany). The reverse transcription reactions were performed with a cDNA reverse transcription kit (#4368814, Applied Biosystems, USA) using a PCR system (Gene Amp 9700, Applied Biosystems). Real-time PCR amplification was set up using TaqMan gene expression assays for human ALOX12 (PPH05791C, Qiagen), ALOX15 (Hs00609608_m1), ALOX15B (Hs00153988_m1) and human HPRT1 (Hs99999909_m1) in combination with Universal PCR master mix (#4324018, Applied Biosystems) and performed for 40 cycles on an ABI Prism 7900 HT sequence detection system (Applied Biosystems). We analyzed PCR data using the comparative CT method [15]. Target gene mRNA expression levels were normalized to HPRT1 mRNA expression.

### Immunohistochemistry

Immunohistochemical staining was performed on 4-μm serial cryosections of frozen cardiac tissue from the right atrium. Immunocytochemistry was performed on formalin-fixed human cardiomyocytes and cardiac endothelial cells cultured under normoxic or hypoxic conditions for 6 h. Mouse monoclonal antibodies against ALOX15 (ab 119774, 1:100, Abcam) in phosphate buffered saline (PBS) with 1% bovine serum albumin (BSA), with isotype control, mouse IgG 2b (GR128531-1, Abcam). Goat anti-mouse IgG (H+L) Alexa Fluor 594 (A11032, 1:200, Molecular Probes, USA) in PBS with 1% BSA was used as secondary antibody. Antibody staining was examined with a Zeiss Axioplan 2 Imaging microscope.

### Immunoblots

Human cardiomyocytes and cardiac endothelial cells were incubated under normoxic (21% oxygen) or hypoxic (1% oxygen) conditions for 6 h. Total cellular lysates were prepared using RIPA buffer supplemented with a mammalian protease inhibitor cocktail (Sigma-Aldrich). Immunoblots were prepared as described [3]. The membranes were probed with anti-ALOX15 antibody (ab 119774, Abcam) or anti-β-actin (A5441, Sigma-Aldrich). The relative intensity of the protein bands was quantified using the ImageLab software (BioRad).

### HETE analyses

15-HETE levels in heart tissue and serum were analyzed by liquid chromatography-mass spectrometry (LC-MS) analysis on an Agilent 6490 LC-MS System (Agilent Technologies, Waldbronn, Germany). Heart tissue was homogenized and extracted using Precellys ^®^ 24 (Bertin Technologies, France) and Mixer-Mill MM 400 (Retsch, Haan, Germany) equipment. Frozen heart tissue was transferred into 2 ml Sarstedt tubes containing 6 ceramic beads. Deuterated internal standard (15-HETE-d8 (Cayman Chemical, Ann Arbor, Michigan), 5 ng/sample) was added followed by 200 μl PBS, 100 μl 1% formic acid in water and 1 ml methyl-tertbutyl ether (MTBE). Samples were homogenized using Precellys 24 (6× 20 s) and lipids were then extracted using Mixer Mill for 10 min followed by centrifugation at 1500*g* for 5 min. The organic phase was transferred to new tubes and the extraction with MTBE was repeated twice. After an additional extraction with methanol (1 ml) and heptane (3 ml), the methanol phase was evaporated to dryness and reconstituted in 100 μl 50% methanol/water.

Serum was analyzed by LC-MS after extraction. Briefly, 100 μl serum was mixed with 230 μl water, 750 μl MTBE, 50 μl 1% formic acid, and 80 μl internal standard (20 ng/ml 15-HETE-d8 in 50% methanol). The lipid extraction was performed twice for 15 min at room temperature. The organic phase was separated (1500*g*, 5 min), evaporated under nitrogen, and the residue was reconstituted in 100 μl of the mobile phase consisting of 50% methanol/water.

Ten microliters of the tissue or serum extracts were injected into an LC-MS system. LC-MS-analysis was performed on a 150 mm Kinetex C18 column with 2.6 μm particles using an acetonitrile gradient capable of separating most eicosanoids. The triple quadrupole mass spectrometer was operated in multiple reaction monitoring mode using negative electrospray ionization.

15-HETE levels in cell medium and cell lysates were measured by ELISA (Abcam) after incubating the cells for 6 h in hypoxic (1% oxygen) or normoxic (21% oxygen) conditions. For the cell medium experiments, 5 μmol/L of calcium ionophore (A23187, Sigma-Aldrich) was added for the last 20 min of incubation. To prepare cell lysates, cardiomyocytes were harvested on ice in cold H_2_O and immediately frozen at -80° before 15-HETE analysis according to the manufacturer’s protocol.

### ROTEM analysis

Whole blood clot formation was assessed with rotational thromboelastometry (ROTEM; Pentapharm, Munich, Germany). Technical details and evaluation of the ROTEM method have been reported earlier [16]. Blood from healthy volunteers was collected in citrate tubes and all ROTEM analyses were performed within 3 h of blood collection [17].

Activation of clot formation was performed using intrinsic (INTEM), extrinsic (EXTEM) and fibrin-based (FIBTEM) assays according to the manufacturer´s protocol in the presence or absence of 15-HETE (Cayman Chemicals) dissolved in dimethylsulfoxide (DMSO) (Sigma). DMSO was used as control. Clotting time, clot formation time, maximum clot firmness, and alpha-angle were measured.

### Statistics

Data were plotted as mean and SEM unless stated otherwise. Levels of significance for differences between group means were determined with Student´s t-test, Mann-Whitney test or one-way ANOVA with Tukey´s multiple comparisons test. GraphPad Prism version 6 (GraphPad Software, San Diego California, www.graphpad.com) was used for calculation of all statistics. *P* values < 0.05 (two-sided) were considered statistically significant.

## Results

### Higher ALOX15 expression and 15-HETE concentrations in human ischemic heart tissue

**Fig 1:**
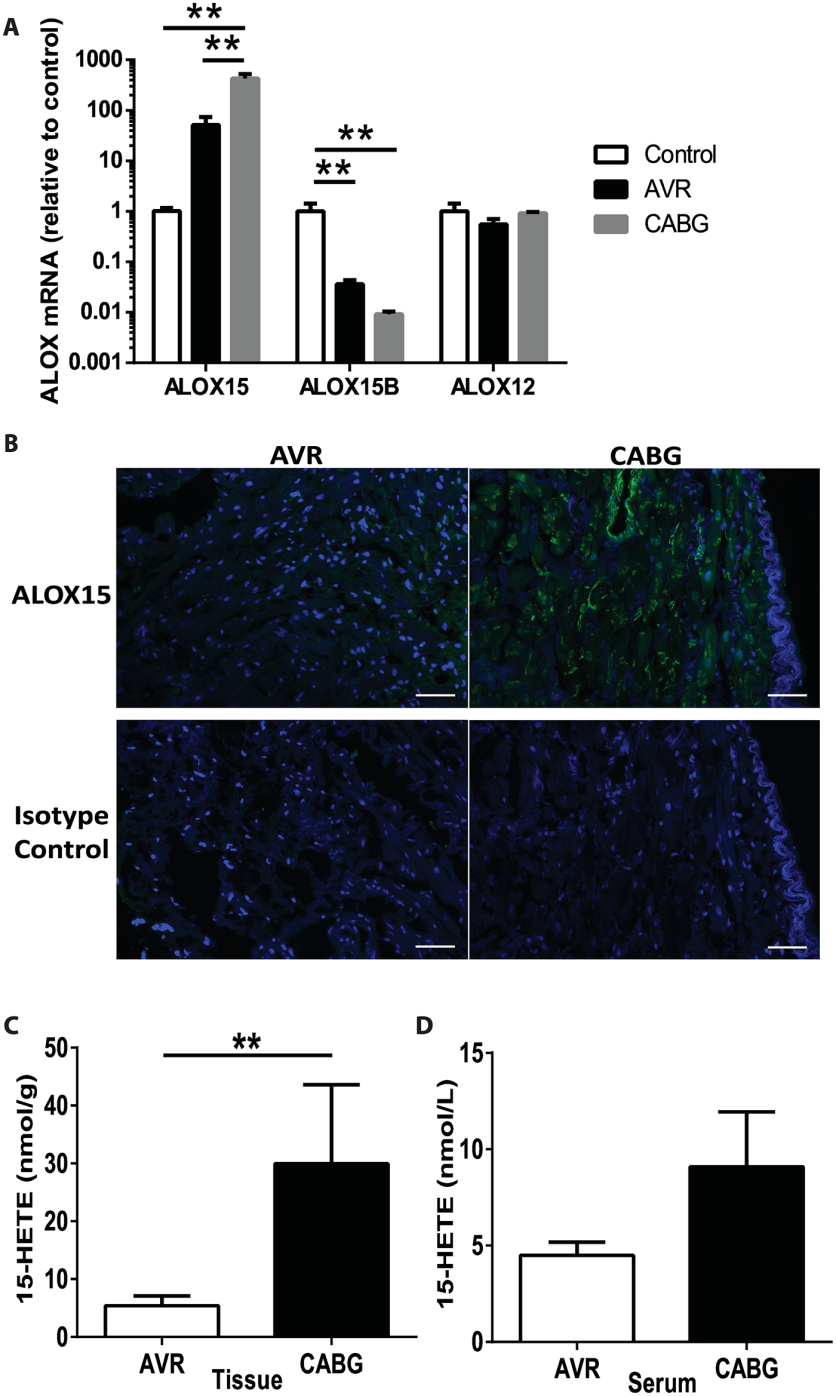
Increased expression of ALOX15 and 15-HETE levels in human ischemic heart biopsies. (**A**) ALOX15, ALOX15B and ALOX12 mRNA expression n (relative to the control for their respective gene) in right atrium from control adults (n = 3) and from patients undergoing AVR (n = 5) or CABG (n = 5) surgery. (**B**) Serial sections of biopsies from right atrium of patients undergoing AVR (left panels) or CABG (right panels) surgery. Sections were stained with DAPI (blue) and antibodies against ALOX15 (green, upper panels) or ALOX15 isotype control (green, lower panels). Scale bar = 50 μm. (**C)** 15-HETE levels in right atrial tissue samples from patients undergoing AVR (n = 5) or CABG (n = 5) surgery. Data are expressed as mean ± SEM. (**D)** 15-HETE levels in serum from patients undergoing AVR (n = 5) or CABG (n = 5) surgery. Data are expressed as mean ± SEM. (**A**) One way ANOVA with Tukey’s multiple comparisons test (** *p* < 0.01). (**C**) Mann-Whitney test (** *p* = 0.0079).

Patient characteristics are shown in Table 1. There were no clear differences between patients undergoing CABG (to treat ischemic heart disease) or AVR (who did not have ischemic heart disease) with regard to the degree of heart failure as measured by echocardiography before surgery, and none of the patients was suffering from severe end-stage heart failure (Table 1). Gene expression analysis of tissue samples from the right atrium of patients undergoing CABG or AVR or controls showed that expression of ALOX15 was higher in ischemic compared with non-ischemic tissue and control tissue (Fig 1A). In agreement, immunohistochemical staining showed higher levels of ALOX15 protein in biopsies collected during CABG surgery compared with biopsies collected during AVR surgery (Fig 1B). By contrast, expression levels of ALOX15B were lower in both AVR and CABG groups compared with controls and ALOX12 expression was similar in biopsies from all the groups (Fig 1A).

**Table 1:** Patient characteristics.

	AVR	CABG
Age, years	68–81	68–81
Sex, M/F	3/2	4/1
NYHA class, I/II/III/IV	3/1/1/0	4/1/0/0
LVEF	40–75%	40–60%
Angiography		
Normal	5	0
2-vessel stenosis	0	1
3-vessel stenosis	0	4

AVR, arterial valve replacement; CABG, coronary arterial bypass grafting; NYHA, New York Heart Association functional classification; LVEF, left ventricle ejection fraction

To investigate whether 15-HETE levels were increased in patients with ischemic heart disease, we assessed 15-HETE concentrations in tissue samples from the right atrium and serum from patients undergoing CABG or AVR. 15-HETE levels were significantly higher in biopsies from CABG patients than in biopsies from AVR patients (Fig 1C). In serum from the same patients, we found a trend towards increased levels of 15-HETE in the CABG patients compared with AVR patients (Fig 1D).

### Higher ALOX15 expression and 15-HETE concentrations in human cardiomyocytes cultured under hypoxic conditions

**Fig 2:**
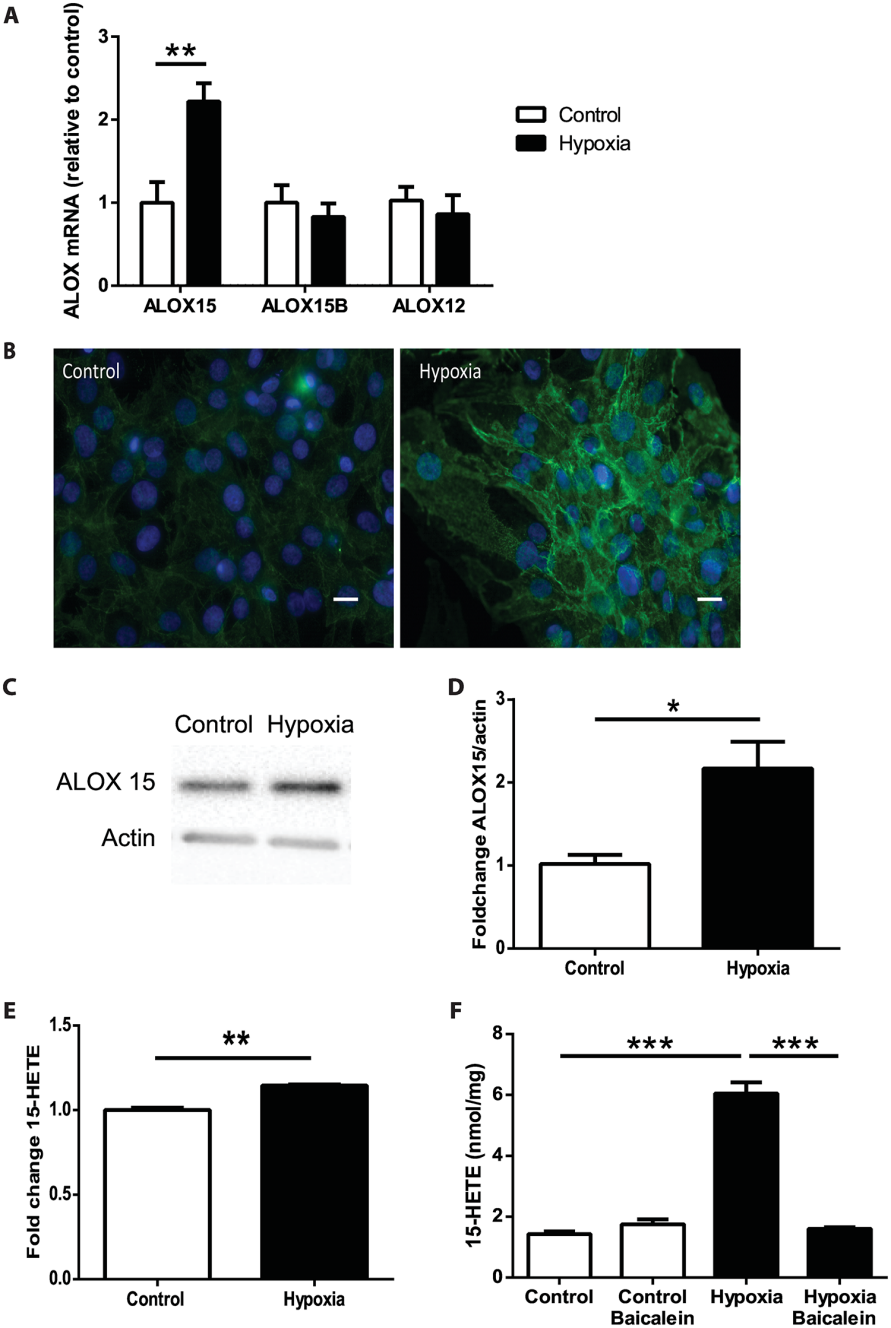
Increased expression of ALOX15 and 15-HETE levels in hypoxic human cardiomyocytes. (**A**) ALOX15, ALOX15B and ALOX12 mRNA expression (relative to the control for their respective gene) in human cardiomyocytes derived from induced pluripotent stem cells incubated under normoxic (21% oxygen; control) or hypoxic conditions (1% oxygen) for 6 h (n = 3). **(B)** Immunocytochemical staining of human cardiomyocytes with antibody against ALOX15 (green) and DAPI (blue) in cells incubated in normoxia (control) or hypoxia. Scale bar = 40 μm. **(C-D)** Representative immunoblot and quantification of ALOX15 protein in human cardiomyocytes incubated in normoxia (control) or hypoxia (n = 3), **(E)** 15-HETE levels in cell culture media from cardiomyocytes incubated in normoxia (control) or hypoxia. **(F)** 15-HETE levels in lysates from human cardiomyocytes incubated in normoxia (control) or hypoxia with or without baicalein (25 μmol/L) for 6 h (n = 3–6). Data are expressed as mean ± SEM. (**A-D**) Student’s t-test (* *p* < 0.05; ** *p* < 0.01). (**F**) One-way ANOVA with Tukey's multiple comparisons test (*** *p* < 0.001).

To investigate whether ischemia affects ALOX15 expression in cardiomyocytes, we cultured human cardiomyocytes derived from pluripotent stem cells in normoxic (21% oxygen) or hypoxic (1% oxygen) conditions for 6 h. ALOX15 mRNA levels in cardiomyocytes increased almost 3-fold after hypoxic incubation compared with cells incubated in normoxic conditions. No significant change was observed for ALOX15B or ALOX12 mRNA expression (Fig 2A). Immunocytochemical staining and immunoblot analysis of hypoxic human cardiomyocytes showed increased levels of ALOX15 protein compared with cells cultured under non-hypoxic conditions (Fig 2B–2D).

To investigate whether the increased ALOX15 levels were paralleled by increased 15-HETE production, we analyzed the effect of hypoxia on 15-HETE concentrations in cell culture medium and cell lysates of human cardiomyocytes. 15-HETE levels were higher in cell medium from cardiomyocytes incubated under hypoxic compared with normoxic conditions (Fig 2E). Furthermore, 15-HETE levels increased 6-fold in cell lysates of human cardiomyocytes after hypoxic incubation and the hypoxia-induced increase in 15-HETE levels was inhibited when cardiomyocytes were treated with the 12/15-lipoxygenase inhibitor baicalein (Fig 2F).

### Higher ALOX15 expression and 15-HETE concentrations in human cardiac endothelial cells cultured under hypoxic conditions

**Fig 3:**
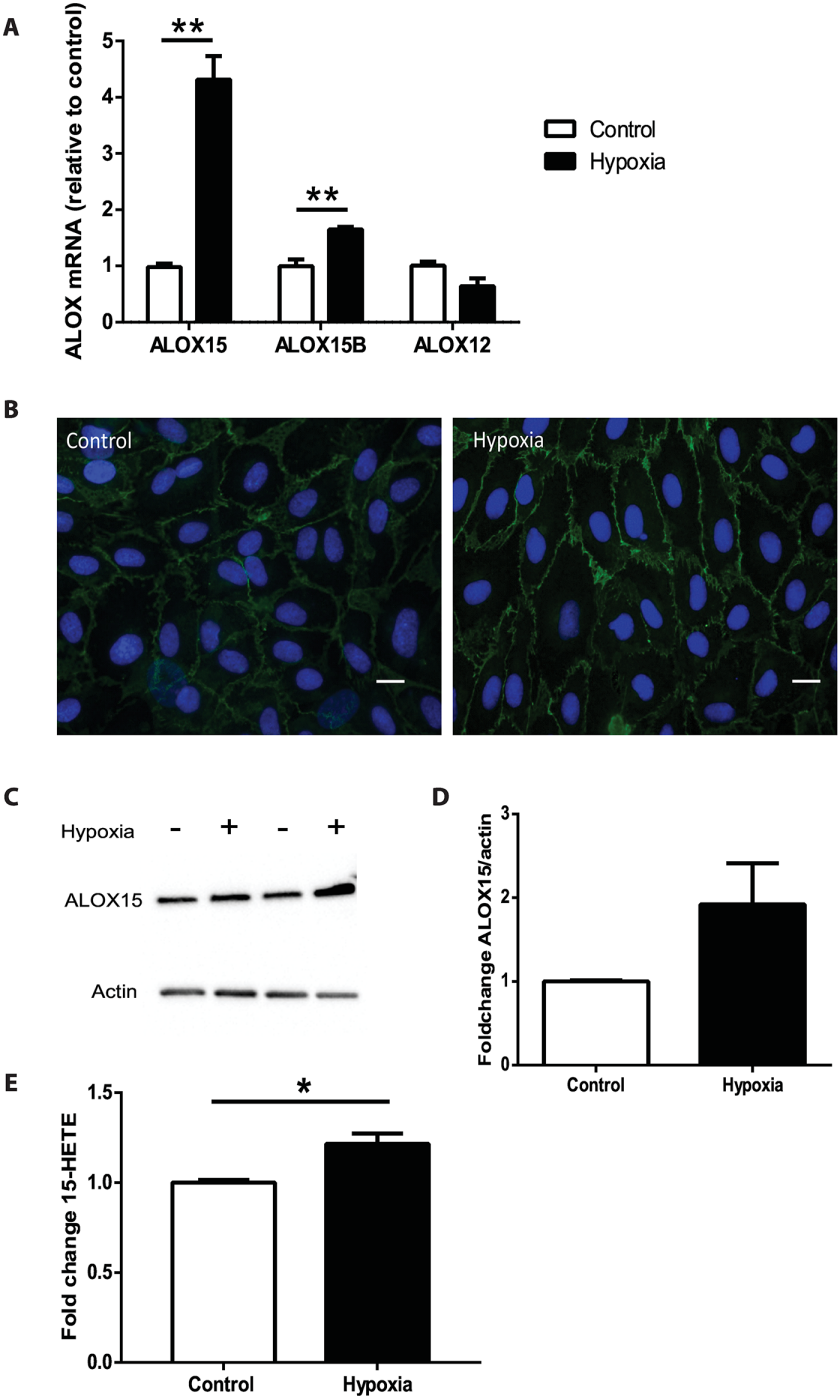
Increased expression of ALOX15 and 15-HETE levels in hypoxic human cardiac endothelial cells. (**A**) ALOX15, ALOX15B and ALOX12 mRNA expression (relative to the control for their respective gene) in human cardiac endothelial cells incubated under normoxic (21% oxygen; control) or hypoxic conditions (1% oxygen) for 6 h (n = 3) (**B**) Immunocytochemical staining of human cardiac endothelial cells with antibody against ALOX15 (green) and DAPI (blue) in cells incubated in normoxia (control) or hypoxia. Scale bar 40 μm. **(C-D)** Representative immunoblot and quantification of ALOX15 protein in human cardiac endothelial cells incubated in normoxia (control) or hypoxia. **(E)** 15-HETE levels in cell culture media from human cardiac endothelial cells incubated in normoxia (control) or hypoxia. Data are expressed as mean ± SEM (*n* = 3). Student’s t-test (* *p* < 0.05; ** *p* < 0.01).

To investigate whether ischemia affects ALOX15 expression in cardiac endothelial cells, we incubated human cardiac endothelial cells in normoxic or hypoxic conditions for 6 h. We showed that ALOX15 and ALOX15B mRNA levels were approximately 4-fold and 1.5-fold higher in cells incubated under hypoxic compared with normoxic conditions. No significant change was observed for ALOX12 mRNA expression after hypoxia compared with normoxia (Fig 3A). Immunocytochemical staining and immunoblot analysis showed increased levels of ALOX15 protein in human cardiac endothelial cells incubated under hypoxic compared with normoxic conditions (Fig 3B, 3C and 3D). ALOX15 staining was typically detected at the cell surface of endothelial cells (Fig 3B).

To investigate whether the upregulated ALOX15 mRNA expression was paralleled by increased 15-HETE production, we analyzed the effect of hypoxia on 15-HETE concentrations in cell culture medium of human cardiac endothelial cells. Levels of 15-HETE increased in medium from human cardiac endothelial cells after hypoxic incubation compared to cells incubated in normoxic conditions (Fig 3E).

### 15-HETE affects the intrinsic coagulation pathway

We used the ROTEM system to assess the effect of 15-HETE on blood coagulation. The INTEM and EXTEM assays test the intrinsic and extrinsic pathway of coagulation respectively. The FIBTEM assay tests blood clot firmness, without any impact of platelets. In the INTEM assay, clotting time was significantly reduced in the presence of 15-HETE as compared to control samples (Table 2). In the EXTEM assay, clot formation time was significantly reduced and maximum clot firmness and the α-angle were significantly increased in the presence of 5 nmol/l 15-HETE (Table 2). In the FIBTEM assay, clotting time was reduced and the α-angle was increased in the presence of 5 nmol/l 15-HETE as compared to control samples (Table 2). These data show that blood clots form faster in the presence of 15-HETE.

**Table 2:** Rotational thromboelastometry data.

ROTEM test		Control	15-HETE (5 nmol/L)	15-HETE (10 nmol/L)	*p* ANOVA
**INTEM**	CT (s)	165±3.7	157±3.7**	153±3.5***	<0.0001
	CFT (s)	80±10.5	77±9.9	79±10.1	0.5032
	MCF (mm)	61±1.3	61±1.4	62±2.4	0.6897
	α-angle (°)	76±1.1	76±1.1	76±1.3	0.7692
**EXTEM**	CT (s)	58±2.1	55±1.3	53±2.25	0.2267
	CFT (s)	92±9.8	84±9.3**	88±8.3	0.0093
	MCF (mm)	61±1.5	62±1.5**	61±1.5	0.0022
	α-angle (°)	72±1.7	74±1.6*	73±1.4	0.0300
**FIBTEM**	CT (s)	57±2.1	50±1.4**	53±1.8	0.0020
	MCF (mm)	13±1.0	14±1.2	13±1.1	0.5703
	α-angle (°)	61±3.6	69±1.9*	66±2.3	0.0202

CT, clotting time, CFT, clot formation time, MCF, maximum clot firmness, and α-angle. Data are from ten blood donors and expressed as mean ±SEM. ANOVA and Tukey's multiple comparisons test,

*p<0.05,

** p<0.01,

***p<0.001 versus control

## Discussion

In this study, we showed that expression of ALOX15 (but not ALOX15B or ALOX12) and 15-HETE levels were substantially higher in myocardial tissue from patients undergoing heart surgery for ischemic heart disease compared with myocardial tissue from those undergoing AVR surgery. The increased expression of ALOX15 mRNA in ischemic heart disease is in agreement with our earlier results [2] but we had not previously investigated ALOX15B or ALOX12 expression in ischemic heart tissue. We also observed a trend toward higher 15-HETE levels in the serum from the patients with ischemic heart disease. In addition, we showed that hypoxia increased the expression of ALOX15 and the production of 15-HETE from human cardiomyocytes and cardiac endothelial cells. Importantly, the ALOX15 inhibitor baicalein inhibited the hypoxia-induced increase in 15-HETE levels from human cardiomyocytes. Thus, we propose that increased ALOX15 expression in heart tissue under ischemic conditions may lead to increased production of 15-HETE, and thus may contribute to the pathogenesis of ischemic heart disease.

A recent analysis of the cellular composition of the heart showed that cardiomyocytes and cardiac endothelial cells account for 30% and 60% of the total number of cells, respectively, and fibroblasts, pericytes, smooth muscle cells and leucocytes account for the remaining 10% [12]. However, because of their large size, cardiomyocytes constitute about 70–90% of the total volume in the normal adult heart [18]. Cardiomyocytes are located close to cardiac endothelial cells within the capillary network, and functional crosstalk between the different cardiac cell types is crucial to maintaining heart function. Here we showed that both cardiomyocytes and cardiac endothelial cells could potentially contribute to the increased 15-HETE levels observed in ischemic heart tissue. We have previously shown that hypoxia also increases ALOX15 in human smooth muscle cells [4], and thus this cell type could also contribute to the increased 15-HETE levels in the ischemic human heart tissue.

We and others have previously shown that the ALOX15/15-HETE pathway promotes platelet activation and vascular thrombosis [9–11]. Here we used three ROTEM assays (INTEM, EXTEM and FIBTEM) to investigate the effect of 15-HETE (at concentrations similar to those in serum from patients with ischemic heart disease) on clot formation in human whole blood. We showed that 15-HETE decreased the INTEM clotting time, indicating that clots are formed faster in the presence of 15-HETE, and increased the EXTEM maximum clot firmness, a measure of the maximal viscoelastic strength of the clot. We also observed an accelerated FIBTEM clotting time in the presence of 15-HETE, suggesting that 15-HETE increases the fibrin formation [19]. Studies of the composition of human coronary thrombi have shown that fibrin content positively correlates with the length of time of ischemia [20]. Platelet aggregation measurements are used to predict the occurrence of thrombotic events and increased platelet aggregation has been associated with cardiovascular events [21]. Clinical settings associated with hypercoagulability detected by thromboelastography include ischemic heart disease [22] and cancer [23], and it has been suggested that ROTEM can be used to identify patients with an increased risk of thrombotic events following surgery [24]. Our data demonstrate evidence of accelerated clot formation in the presence of 15-HETE, which theoretically may lead to hypercoagulability of human blood. ALOX15-mediated 15-HETE synthesis may therefore be of biological importance in ischemic heart disease.

In addition to the increased ALOX15 levels observed in hypoxic human cardiomyocytes and cardiac endothelial cells in the present study and in human smooth muscle cells in an earlier study [4], we have previously shown that ALOX15B protein levels are increased in macrophages from carotid atherosclerotic plaques and that hypoxic macrophages express active ALOX15 leading to 15-HETE production [25]. Thus, our data combined suggest that the local environment within vessels or atherosclerotic plaques could contribute to locally increased 15-HETE and thus potentiate thrombus formation.

Taken together, our results are consistent with a role for ALOX15/15-HETE signaling in ischemic heart disease. However, future studies are required to determine whether the increased 15-HETE production in ischemic heart tissue contributes to thrombosis and the potential for ALOX15 as a therapeutic target for myocardial infarction.

## Supporting Information

S1 Fig Immunocytochemistry staining of human cardiomyocytes derived from induced pluripotent stem cells: cardiac troponin (red), F-actin (green) and DAPI (blue). (TIF)

S2 Fig Immunocytochemistry staining of human cardiac endothelial cells: endothelial cell marker CD31 (green) and DAPI (blue). (TIF)
