# Supplementary figures and images for: The Arachidonate 15-Lipoxygenase Enzyme Product 15-HETE Is Present in Heart Tissue from Patients with Ischemic Heart Disease and Enhances Clot Formation
Source: PLoS One. 2016 Aug 23;11(8):e0161629. doi: 10.1371/journal.pone.0161629 (PMC4994938; doi:10.1371/journal.pone.0161629)

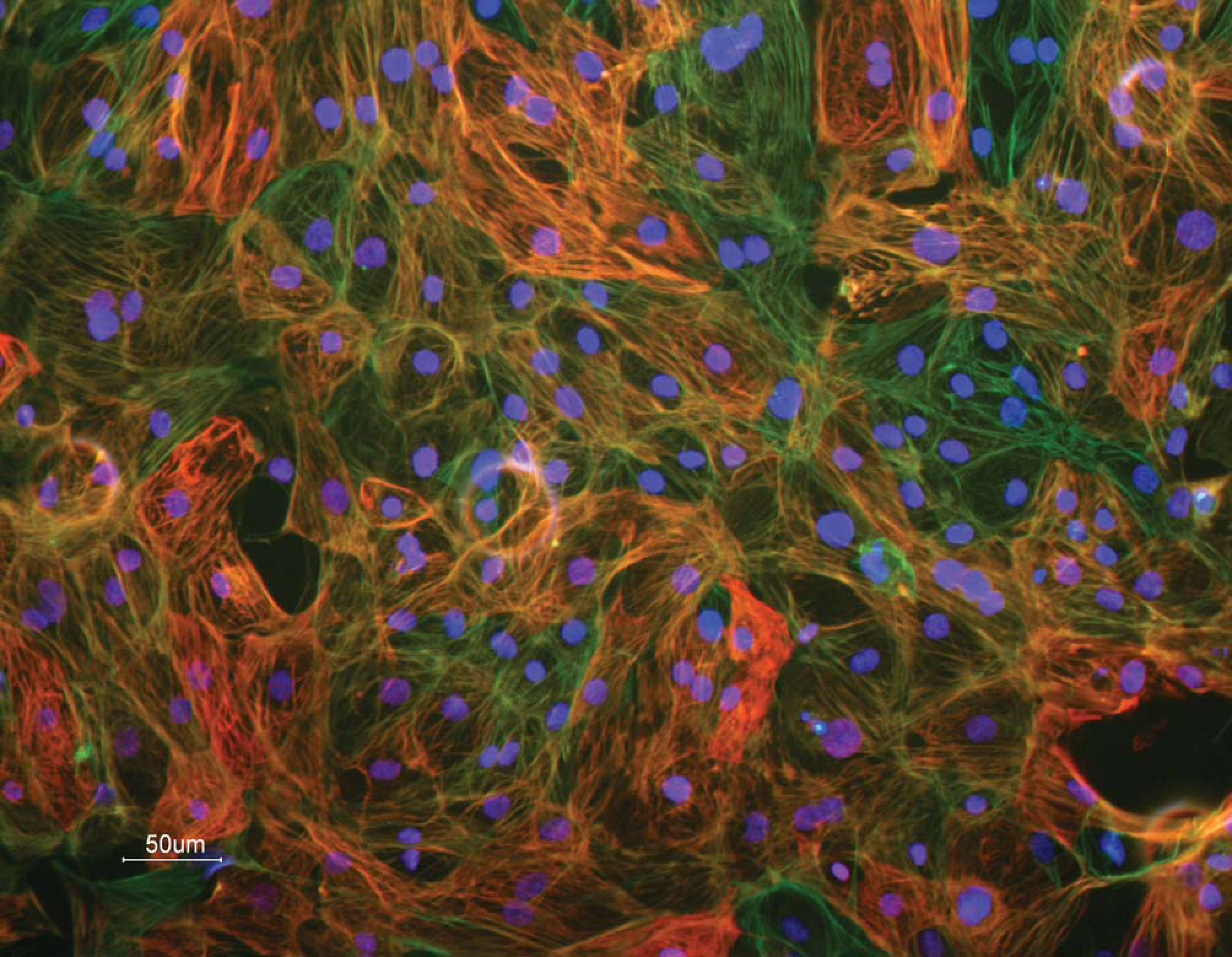

Supplement: S1 Fig — (TIF) [file pone.0161629.s001.tif]

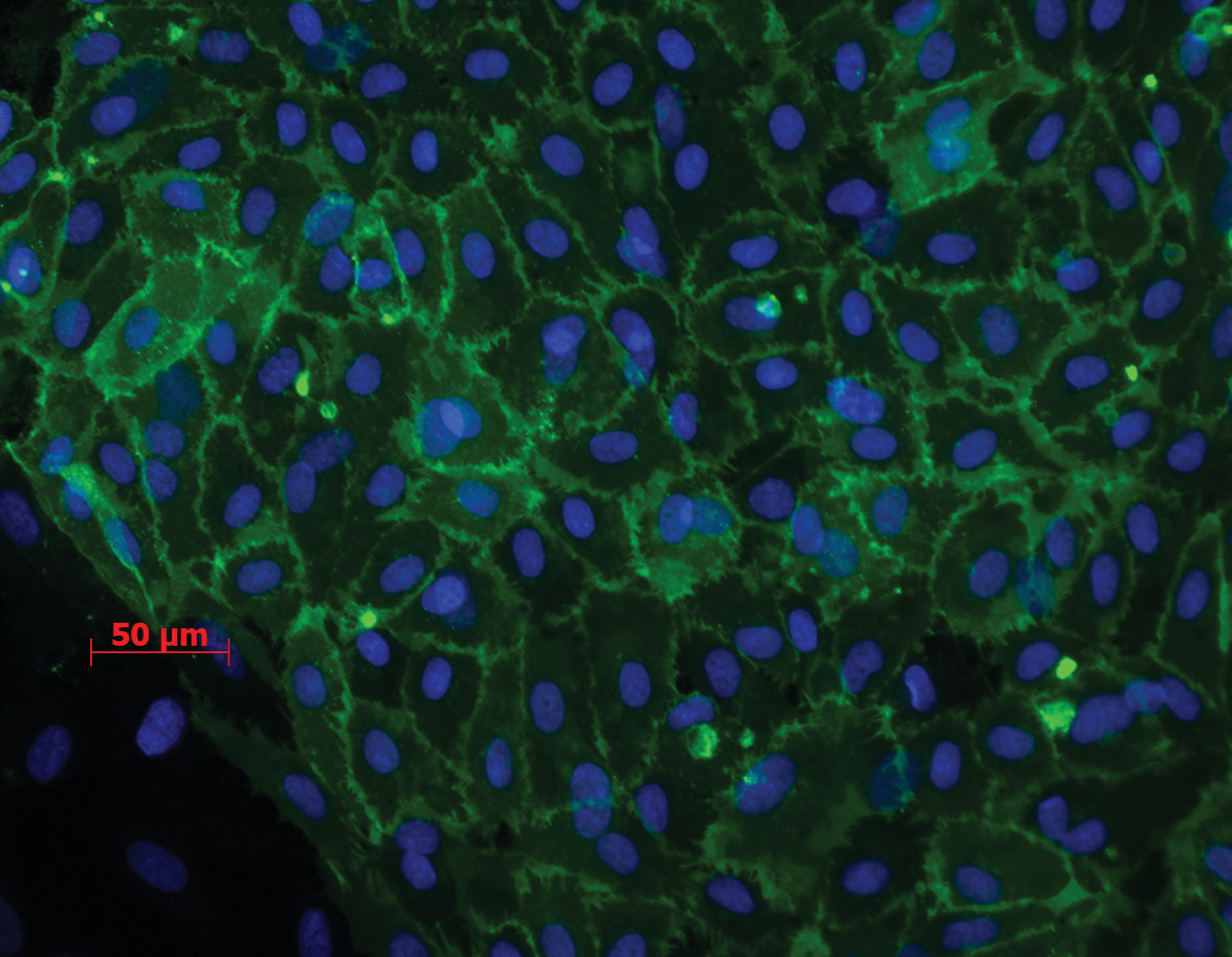

Supplement: S2 Fig — (TIF) [file pone.0161629.s002.tif]
